# A case of PTSD presenting with psychotic symptomatology: a case report

**DOI:** 10.1186/1757-1626-1-352

**Published:** 2008-11-25

**Authors:** Georgios D Floros, Ioanna Charatsidou, Grigorios Lavrentiadis

**Affiliations:** 1 2nd Department of Psychiatry, Psychiatric Hospital of Thessaloniki, 196 Langada str., 564 29 Thessaloniki, Greece

## Abstract

A male patient aged 43 presented with psychotic symptomatology after a traumatic event involving accidental mutilation of the fingers. Initial presentation was uncommon although the patient responded well to pharmacotherapy. The theoretical framework, management plan and details of the treatment are presented.

## Background

Recent studies have shown that psychotic symptoms can be a hallmark of post-traumatic stress disorder [1,2]. The vast majority of the cases reported concerned war veterans although there were sporadic incidents involving non-combat related trauma (somatic or psychic). There is a biological theoretical framework for the disease [3] as well as several psychological theories attempting to explain cognitive aspects [4].

## Case presentation

A male patient, aged 43, presented for treatment with complaints tracing back a year ago to a traumatic work-related event involving mutilation of the distal phalanges of his right-hand fingers. Main complaints included mixed hallucinations, irritability, inability to perform everyday tasks and depressive mood. No psychic symptomatology was evident before the event to him or his social milieu.

## Mental state examination

The patient was a well-groomed male of short stature, sturdy build and average weight. He was restless but not agitated, with a guarded attitude towards the interviewer. His speech pattern was slow and sparse, his voice low. He described his current mood as 'anxious' without being able to provide with a reason. Patient appeared dysphoric and with blunted affect. He was able to maintain a linear train of thought with no apparent disorganization or irrational connections when expressing himself. Thought content centred on his amputated fingers with a semi-compulsive tendency to gaze to his (gloved) hand. The patient was typically lost in ruminations about his accident with a focus on the precise moment which he experienced as intrusive and affectively charged in a negative and painful way. He could remember wishing for his fingers to re-attach to his hand almost as the accident took place. A trigger in his intrusive thoughts was the painful sensation of neuropathic pain from his half-mutilated fingers, an artefact of surgery.

He denied and thoughts of harming himself and demonstrated no signs of aggression towards others. Hallucinations had a predominantly depressive and ego-dystonic character. He denied any perceptual disturbances at the time of the examination. Their appearance was typically during nighttime especially in the twilight. Initially they were visual only, involving shapes and rocks tumbling down towards the patient, gradually becoming more complex and laden with significance. A mixed visual and tactile hallucination of burning rain came afterwards while in the time of examination a tall stranger clad in black and raiding a tall steed would threaten and ridicule the patient. He scored 21 on a MMSE with trouble in the attention, calculation and recall categories. The patient appeared reliable and candid to the extent of his self-disclosure, gradually opening up to the interviewer but displayed a marked difficulty on describing his emotions and memories of the accident, apparently independent of his conscious will. His judgement was adequate and he had some limited Insight into his difficulties, hesitantly attributing them to his accident.

He was married and a father of three (two boys and a girl aged 7–12) He had no prior medical history for mental or somatic problems and received no medication. He admitted to occasional alcohol consumption although his relatives confirmed that he did not present addiction symptoms. He had some trouble making ends meet for the past five years. Due to rampant unemployment in his hometown, he was periodically employed in various jobs, mostly in the construction sector. One of his children has a congenital deformity, underwent several surgical procedures with mixed results and, before the time of the patient's accident, it was likely that more surgery would be forthcoming. The patient's father was a proud man who worked hard but reportedly was victimized by his brothers, they reaping the benefits of his work in the fields by manipulating his own father. He suffered a nervous breakdown attributed to his low economic status after a failed economic endeavour ending in him being robbed of the profits, seven years before the accident. There was no other relevant family history.

Before the accident the patient was a lively man, heavily involved as a participant and organizer in important local social events from a young age. He was respected by his fellow villagers and felt his involvement as a unique source of pride in an otherwise average existence. Prior to his accident, the patient was repeatedly promised a permanent job as a labourer and fate would have it that his appointment was supposedly approved immediately after the accident only to be subsequently revoked. He viewed himself as an exploited man in his previous jobs, much the same way his father was, while he harboured an extreme bitterness over the unavailability of support for his long-standing problems. His financial status was poor, being in sick-leave from his previous job for the last four months following the accident and hoping to receive some compensation. Although his injuries were considered insufficient for disability pension he could not work to his full capacity since the hand affected was his primary one and he was a manual labourer.

Given that the patient clearly suffered a high level of distress as a result of his hallucinatory experiences he was voluntary admitted to the 2nd Psychiatric Department of the Aristotle University of Thessaloniki for further assessment, observation and treatment. A routine blood workup was ordered with no abnormalities. A Rorschach Inkblot Test was administered in order to gain some insight into patient's dynamics, interpersonal relations and underlying personality characteristics while ruling out any malingering or factitious components in the presentation as suggested in Wilson and Keane [5]. Results pointed to inadequate reality testing with slight disturbances in perception and a difficulty in separating reality from fantasy, leading to mistaken impressions and a tendency to act without forethought in the face of stress. Uncertainty in particular was unbearable and adjustment to a novel environment hard. Cognitive functions (concentration, attention, information processing, executive functions) were impaired possibly due to cognitive inability or neurological disease. Emotion was controlled with a tendency for impulsive behaviour; however there was difficulty in processing and expressing emotions in an adaptive manner. There were distinct patterns of aggression and anger towards others but expressing those patterns was avoided, switching to passivity and denial rather than succumbing to destructive urges or mature competitiveness. Self-esteem was low with feelings of inferiority and inefficiency.

A neurological examination revealed a left VI cranial nerve paresis, reportedly congenital, resulting in diplopia while gazing to the extreme left, which did not significantly affect the patient. The patient had a chronic complaint of occasional vertigo, to which he partly attributed his accident, although the symptoms were not of a persisting nature.

Initial diagnosis at this stage was 'Psychotic disorder NOS' and pharmacological treatment was initiated. An MRI scan of the brain with gadolinium contrast was ordered to rule out any focal neurological lesions. It was performed fifteen days later and revealed no abnormalities.

**Figure 1:**
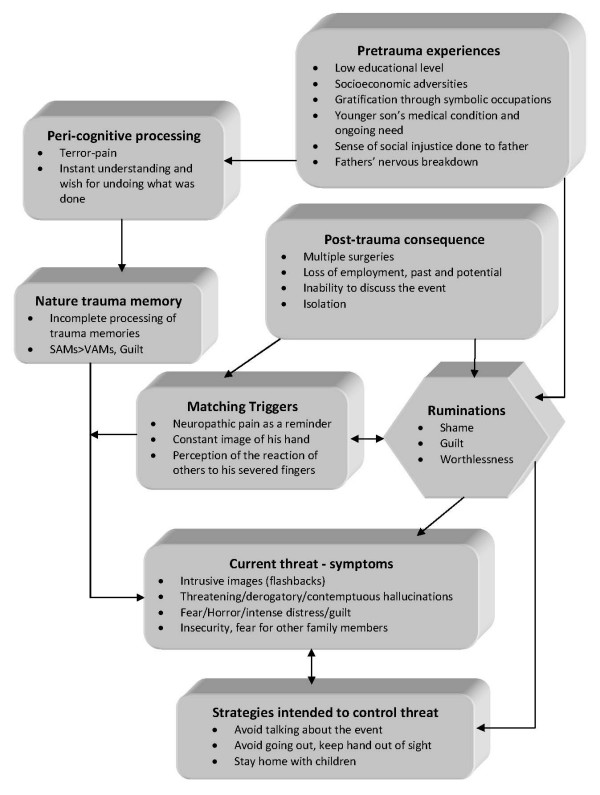
**Case formulation – (Persistent PTSD, adapted from Ehlers and Clark **[6]**)**. Case formulation following the persistent PTSD model of Ehlers and Clark [6]. It is suggested that the patient is processing the traumatic information in a way which a sense of immediate threat is perpetuated through negative appraisals of trauma or its consequences and through the nature of the traumatic experience itself. Peri-traumatic influences that operate at encoding, affect the nature of the trauma memory. The memory of the event is poorly elaborated, not given a complete context in time and place, and inadequately integrated into the general database of autobiographical knowledge. Triggers and ruminations serve to re-enact the traumatic information while symptoms and maladaptive coping strategies form a vicious circle. Memories are encoded in the SAM rather than the VAM system, thus preventing cognitive re-appraisal and eventual overcoming of traumatic experience [4].

Patient was placed on ziprasidone 40 mg bid and lorazepam 1 mg bid. He reported an immediate improvement but when the attending physician enquired as to the nature of the improvement the patient replied that in his hallucinations he told the tall raider that he now had a tall doctor who would help him and the raider promptly left (sic). Apparently, the random assignment of a strikingly tall physician had an unexpected positive effect. Ziprasidone gradually increased to 80 mg bid within three days with no notable effect to the perceptual disturbances but with the development of akathisia for which biperiden was added, 1 mg tid. Duloxetine was added, 60 mg once-daily, in a hope that it could have a positive effect to his mood but also to this neuropathic pain which was frequent and demoralising. The patient had a tough time accommodating to the hospital milieu, although the grounds were extended and there was plenty of opportunity for walks and other activities. He preferred to stay in bed sometimes in obvious agony and with marked insomnia. He presented a strong fear for the welfare of his children, which he could not reason for. Due to the apparent inability of ziprasidone to make a dent in the psychotic symptomatology, medication was switched to amisulpride 400 mg bid and the patient was given a leave for the weekend to visit his home. On his return an improvement in his symptoms was reported by him and close relatives, although he still had excessive anxiety in the hospital setting. It was decided that his leave was to be extended and the patient would return for evaluation every third day. After three appointments he had a marked improvement, denied any psychotic symptoms while his sleep pattern improved. A good working relationship was established with his physician and the patient was with a schedule of follow-up appointments initially every fifteen days and following two months, every thirty days. His exit diagnosis was "Psychotic disorder Not Otherwise Specified – PTSD". He remained asymptomatic for five months and started making in-roads in a cognitively-oriented psychotherapeutic approach but unfortunately further trouble befell him, his wife losing a baby and his claim to an injury compensation rejected. He experienced a mood loss and duloxetine was increased to 120 mg per day to some positive effect. His status remains tenuous but he retains a strong will to make his appointments and work with his physician. A case conceptualization following a cognitive framework [6] is presented in Figure 1.

## Discussion

The value of a specialized formulation is made clear in complex cases as this one. There is a relationship between the pre-existing cognitive schemas of the individual, thought patterns emerging after the traumatic event and biological triggers. This relationship, best described as a maladaptive cognitive processing style, culminates into feelings of shame, guilt and worthlessness which are unrelated to similar feelings, which emerge during trauma recollection, but nonetheless acts in a positive feedback loop to enhance symptom severity and keep the subject in a constant state of psychotic turmoil. Its central role is addressed in our case formulation under the heading "ruminations" which best describes its ongoing and unrelenting character. The "what if" character of those ruminations may serve as an escape through fantasy from an unbearably stressful cognition. Past experience is relived as current threat and the maladaptive coping strategies serve as negative re-enforcers, perpetuating the emotional suffering.

The psychosocial element in this case report, the patient's involvement with a highly symbolic activity, demonstrates the importance of individualising the case formulation. Apparently the patient had a chronic difficulty in expressing his emotions and integrating into his social surroundings, a difficulty counter-balanced somewhat with his involvement in the local social events which gave him not only a creative way out from any emotional impasse but also status and recognition. His perceived inability to continue with his symbolic activities was not only an indicator of the severity of his troubles but also a stressor in its own right.

## Conclusion

Complex cases of PTSD presenting with hallucinatory experiences can be effectively treated with pharmacotherapy and supportive psychotherapy provided a good doctor-patient relationship is established and adverse medication effects rapidly dealt with. A cognitive framework and a Rorschach test can be valuable in deepening the understanding of individuals and obtaining a personalized view of their functioning and character dynamics. A biopsychosocial approach is essential in integrating all aspects of the patients' history in a meaningful way in order to provide adequate help.

## Patient's perspective

"My life situation can't seem to get any better. I haven't had any support from anyone in all my life. Leaving home to go anywhere nowadays is hard and I can't seem to be able to stay anyplace else for a long time either. Just getting to the hospital [where the follow-up appointments are held] makes me very nervous, especially the minute I walk in. Can't seem to stay in place at all, just keep pacing while waiting for my appointment. I am only able to open up somewhat to my doctor, whom I thank for his support. Staying in hospital was close to impossible; I was very stressed and particularly concerned for my children, not being able to be close to them. I still need to have them near-by. Getting the MRI scan was also a stressful experience, confined in a small space with all that noise for so long. I succeeded only after getting extra medication.

I hope that things will get better. I don't trust anyone for any help any more; they should have helped me earlier."

## Abbreviations

PTSD: stands for 'Post Traumatic Stress Disorder'; VAM: for 'Verbally Accessible Memory'; SAM: for 'Situationally Accessible Memory'

## Competing interests

The authors declare that they have no competing interests.

## Authors' contributions

GF was the attending SHO and the major contributor in writing the manuscript. IC performed the psychological evaluation and Rorschach testing and interpretation. GL provided valuable guidance in diagnosis and handling of the patient. All authors read and approved the final manuscript.

## Consent

Written informed consent was obtained from the patient for publication of this case report and accompanying images. A copy of the written consent is available for review by the Editor-in-Chief of this journal.
